# Characterization of an *ETV6*-*NTRK3* rearrangement with unusual, but highly significant FISH signal pattern in a secretory carcinoma of the salivary gland: a case report

**DOI:** 10.1186/s13000-021-01133-z

**Published:** 2021-08-09

**Authors:** Florian Wagner, Ralf Greim, Kathrin Krebs, Finn Luebben, Arno Dimmler

**Affiliations:** 1 Zytomed Systems GmbH, Berlin, Germany; 2 Medizinische Klinik 2, Hämatologie, Onkologie, Immunologie, Palliativmedizin, St. Vincentius-Kliniken Karlsruhe, ViDia Christliche Kliniken, Karlsruhe, Germany; 3 Institut und Gemeinschaftspraxis für Pathologie, St. Vincentius-Kliniken Karlsruhe, ViDia Christliche Kliniken, Karlsruhe, Germany

**Keywords:** *ETV6-NTRK3* fusion, Salivary gland, Secretory carcinoma, *NTRK3* break apart FISH, Case report

## Abstract

**Background:**

Fusions of neurotrophic tropomyosin receptor kinase genes *NTRK1*, *NTRK2* and *NTRK3* with various partner genes occur in both common and rare tumours and are of paramount predictive value due to the availability of very effective pan-Trk inhibitors like Larotrectinib and Entrectinib. Detection of NTRK fusions is mainly performed by fluorescence in situ hybridization (FISH) and next generation sequencing (NGS). The case described here showed a very unusual, but highly significant FISH signal pattern with an *NTRK3* break apart probe, indicative of a functional *NTRK3* rearrangement.

**Case presentation:**

We describe here the case of a male patient who was originally diagnosed with an adenocarcinoma of the parotid gland without evidence of metastases. After the development of multiple lung metastases, an extensive immunohistochemical and molecular examination of archived tumour tissue including analysis of *NTRK* was performed. *NTRK* expression was detected by immunohistochemistry (IHC) and then comprehensively analysed further by FISH, quantitative reverse transcription PCR (RT-qPCR), and NGS. *NTRK3* break apart FISH showed multiple and very faint single 3′ signals in addition to fusion signals. Quantitative reverse transcription PCR and NGS confirmed an ETV6:exon5-NTRK3:exon15 fusion. Diagnosis was therefore revised to metastatic secretory carcinoma of the salivary gland, and the patient subsequently treated with Larotrectinib, resulting in persisting partial remission.

**Conclusions:**

Our findings underline the importance to be aware of non-canonical signal patterns during FISH analysis for detection of *NTRK* rearrangements. Very faint single 3′ signals can indicate a functional *NTRK* rearrangement and therefore be of high predictive value.

## Background

Fusions of neurotrophic tropomyosin receptor kinase genes *NTRK1*, *NTRK2* and *NTRK3* with various partner genes have been detected in a variety of both common and rare tumour entities [1, 2]. In each case, the 3′ region of *NTRK* coding for the tyrosine kinase (TK) domain is fused to the 5′ region of the partner gene, resulting in ligand-independent, constitutional activation of the TK function [3]. In general, *NTRK* fusions are rare in common cancer types (less than 1%), but highly prevalent (up to or greater than 90%) in some rare cancer entities like secretory breast carcinoma and infantile fibrosarcoma [4]. The protein products encoded by *NTRK1*, *NTRK2*, and *NTRK3*, transmembrane protein kinases TrkA, TrkB and TrkC, respectively, show a high degree of amino acid conservation around their ATP binding pocket, which has favoured the development of several pan-TRK inhibitors [5]. Among these, Larotrectinib [6] and Entrectinib [7] are the first tumour-agnostic TK inhibitors approved by the Food and Drug Administration and the European Medicines Agency and have shown to be very effective against *NTRK* fusion-positive tumours. Therefore, despite low prevalence in common tumours, *NTRK* fusion-testing is now standard of care in patients with locally advanced or metastatic cancer [8].

## Case presentation

**Fig. 1:**
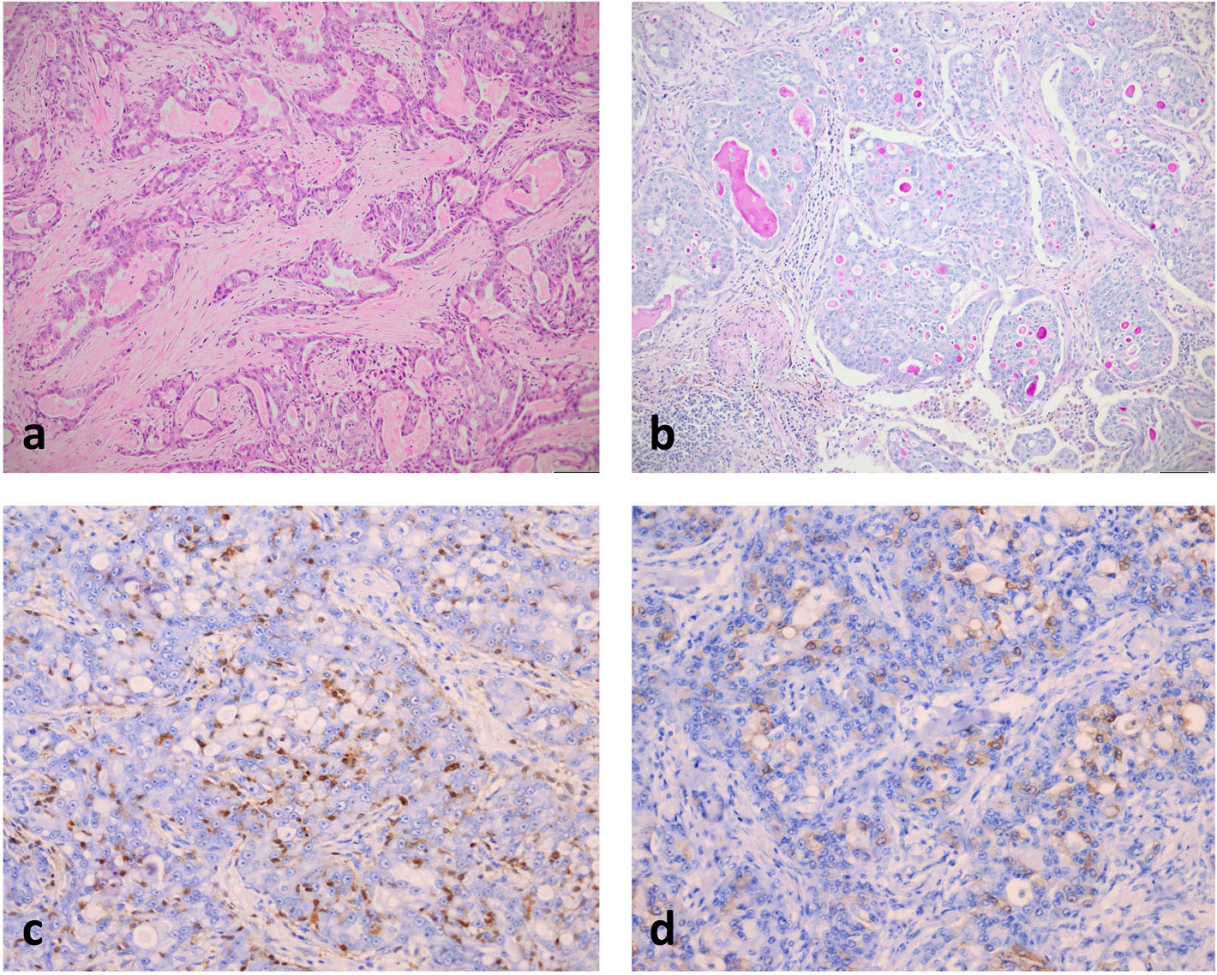
Histological and immunohistochemical features of the tumour. **a** Infiltrates of secretory carcinoma show microcystic to reticular and focally tubular growth and moderate nuclear pleomorphy with accompanying desmoplastic stromal reaction (HE staining). **b** Focal intra- and extracellular PAS-positive mucin production is recognized (PAS staining). **c** Focal expression of S100 protein. **d** Mammaglobin expression in part of tumour cells

**Fig. 2:**
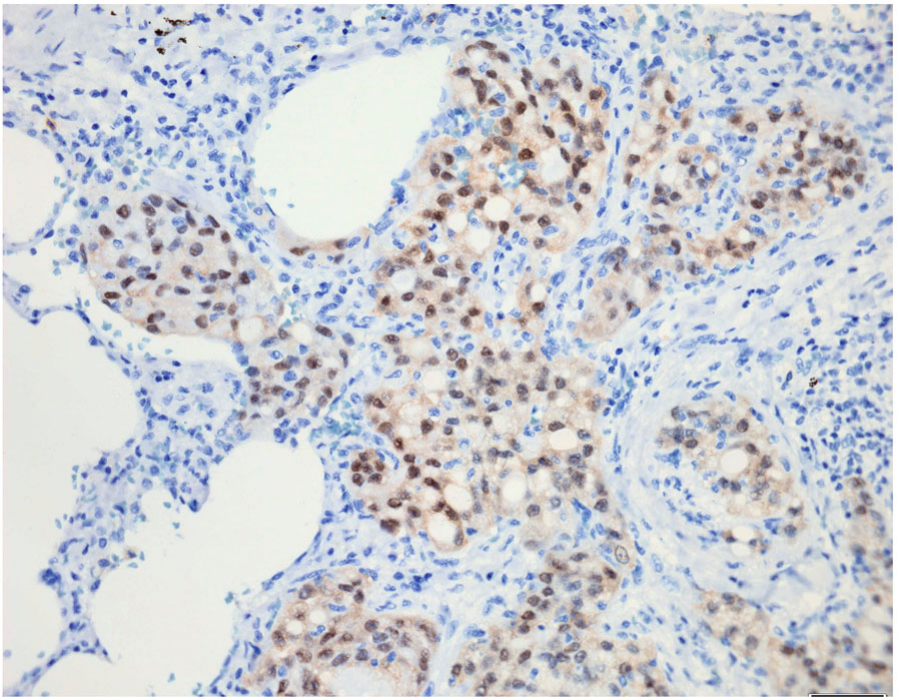
Tumour cells show moderate to strong nuclear staining and weak cytoplasmic staining using an anti-pan Trk antibody

A 38-year-old male patient presented in 2008 with a centrally located tumour of up to 3 cm in diameter of his right parotid gland, which was treated by resection. Macroscopically an ill-defined grey tumour mass was seen, and histologic examination showed microcystic to reticular and focally tubular growth of moderately pleomorphic epitheloid cells with focal intra- and extracellular PAS-positive mucin production (Fig. 1a+b). Muscle infiltration and perineural growth as well as central sclerosis of tumour tissue were recognized. Epidermoid differentiation or presence of goblet cells were not seen. Immunohistochemical examination showed strong expression of cytokeratin 7 and focal weak to moderate expression of S100 protein (Fig. 1c). No expression of alpha-amylase, carcinoembryonic antigen, or smooth muscle actin was detected. Thus after exclusion of main differential diagnoses of acinic cell carcinoma and mucoepidermoid carcinoma, diagnosis of moderately differentiated adenocarcinoma not otherwise specified of parotid gland was made, and neck dissection with removal of 14 right cervical lymph nodes was added without evidence of metastases. In follow-up the patient presented with multiple lung metastases: in 2012 three lung metastases of up to 2.5 cm in diameter in right lung segment 2 and two lung metastases of up to 1.0 cm in diameter in right lung segments 1 und 4 were removed, followed in 2014 by resection of three lung metastases of up to 1.5 cm in diameter in right upper and lower lung lobe. Histologic analysis showed comparable morphology to initial diagnostic sample, and lack of TTF1 expression further confirmed diagnosis of lung metatastases of known parotid gland adenocarcinoma. Finally, in 2017, after palliative chemotherapy four lung metastases of up to 0.6 cm in diameter in left lung segments 1, 2, 7 and 8 were treated by local excision. Because of still progressive pulmonary tumour dissemination and the occurrence of skeletal metastases palliative radiochemotherapy was started and extensive immunohistochemical and molecular examination of archived tumour tissue initiated. Additional IHC stainings showed moderate expression of mammaglobin (Fig. 1d; this marker has only been established in our laboratory since 2015), no expression of DOG1, and moderate to strong nuclear and weak cytoplasmic staining (Fig. 2) using an anti-pan Trk antibody (Clone EPR17341, dilution 1:250, Abcam, Cambridge, United Kingdom).

**Fig. 3:**
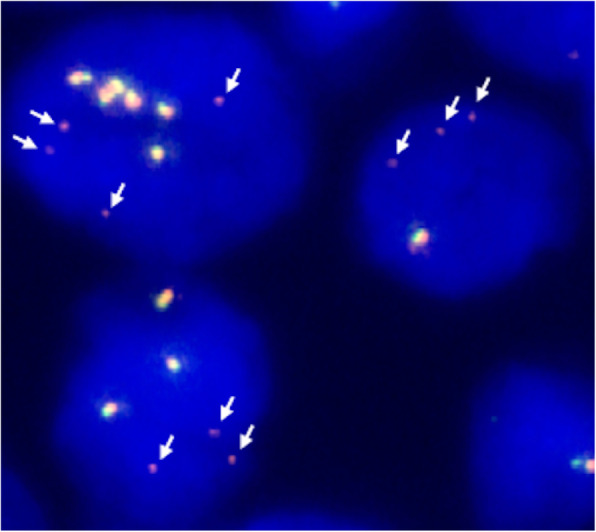
*NTRK3* break apart FISH (original magnification 1000x): Tumour cells show fusion signals and additional faint single orange signals (marked by arrows)

Fluorescence in situ hybridization (FISH) was performed on 3 μm FFPE sections of tumour tissue using break apart probes for *NTRK1*, *NTRK2*, and *NTRK3* (Z-2167, Z-2205, Z-2206; ZytoVision GmbH, Bremerhaven, Germany), each composed of a green-labelled probe for the 5′ part of *NTRK*, and an orange-labelled probe marking the 3′ tyrosine kinase domain. Analysis revealed wild-type fusion signals for *NTRK1* and *NTRK2* but an aberrant pattern for *NTRK3*. In addition to fusion signals, up to four single orange signals were observed in the vast majority of nuclei (Fig. 3). Remarkably, these single orange signals were a lot fainter than orange signals being part of fusion signals.

Next, RNA was isolated (FFPE RNA Kit, Amoy Diagnostics, Xiamen, China) and quantitative reverse transcription PCR performed with a panel comprising 109 different fusion variants for *NTRK1/2/3* (NTRK Gene Fusions Detection Kit, Amoy Diagnostics). This assay uses a pooling strategy, and data analysis showed positive signals for two pools, each representing several different *ETV6-NTRK3* fusion transcripts. Finally, RNA-based next generation sequencing (RNA-Seq) was executed in order to determine exact variants.

RNA-Seq was performed with two different panels independently. The first analysis was conducted using the Archer FusionPlex Comprehensive Thyroid and Lung (CTL) panel (ArcherDX, Boulder, Colorado, USA). Sequencing was done on a MiSeq sequencer (Illumina, San Diego, California, USA), and sequencing data was processed using Archer Analysis 6.2 Site. Archer FusionPlex analysis revealed the presence of three different *ETV6-NTRK3* variants (Table 1), with ETV6:exon5-NTRK3:exon15 as the dominant variant.

**Table 1:** Frequency of three different *ETV6-NTRK3* fusion variants detected by RNA-Seq

Fusion variant	Number of reads	
	Archer panel	AmoyDx panel
ETV6:exon5-NTRK3:exon15	3011	10,349
ETV6:exon5-NTRK3:exon16	10	125
ETV6:exon4-NTRK3:exon15	7	28

Second, a hybridization probe-based library preparation kit targeting 36 genes including *NTRK1/2/3* was used (AmoyDx HANDLE Classic NGS Panel, Amoy Diagnostics). Sequencing was performed on a MiniSeq sequencer (Illumina, San Diego, California, USA), and data analysis resulted in detection of the same three *ETV6-NTRK3* fusion variants as detected by the Archer panel (Table 1). In addition, the relative abundance of these variants was comparable, again with ETV6:exon5-NTRK3:exon15 presenting as the dominant variant.

With *ETV6-NTRK3* fusion confirmed, the patient’s diagnosis was revised to metastatic secretory carcinoma of the salivary gland, and treatment with pan-Trk inhibitor Larotrectinib (100 mg twice daily) initiated. After 8 months of treatment, CT and MRI scans showed partial remission with pulmonary and skeletal metastases of reduced size.

## Discussion and conclusions

We describe here the case of a male patient with an *ETV6*-*NTRK3* fusion positive salivary gland carcinoma. The entity of secretory carcinoma [9] had not been described at the time of initial adenocarcinoma diagnosis (2008) in the present case, and due to just focal S100 expression, it was only suspected later after positive IHC staining results for mammaglobin and pan-Trk. Nowadays secretory carcinoma is an important part of differential diagnostic spectrum after exclusion of similar and more common entities like acinic cell carcinoma (expression of alpha amylase and/or DOG1), mucoepidermoid carcinoma (distinction mainly based on morphology, i.e. epidermoid differentiation and goblet cells), salivary duct carcinoma (expression of androgen receptor and/or overexpression of ERBB2), myoepithelial carcinoma (expression of smooth muscle actin), or polymorphous adenocarcinoma (expression of S100 and mammaglobin possible, no Trk expression) .

In the case presented here the *ETV6-NTRK3* fusion and its effects were broadly characterized by IHC, FISH, RT-qPCR, and finally NGS. While IHC with an anti-pan TRK antibody resulted in a mainly nuclear and weaker cytoplasmic staining typical for this type of fusion [10], the FISH staining pattern was quite exceptional. Multiple very faint single orange signals were observed in addition to fusion signals. This could be explained by loss of a major part of the region - but not the tyrosine kinase domain - targeted by the orange-labelled 3′ probe (about 495 kb) during rearrangement. Most of the 3′ target region is downstream of *NTRK3*, hence its loss would not affect functionality of the tyrosine kinase domain. In conclusion, faint single orange (3′) signals would be the result of a complete loss of the 5′ target region (green signal) in combination with loss of most of the 3′ target region. This scenario would require several double strand breaks, but it is well known from extensively analysed rearrangements like *EML4-ALK* that these can be notably more complex than just involving the minimum of two double strand breaks. E.g., an *EML4-ALK* translocation with at least five different genomic break points and subsequent loss of several non-connected regions has been described [11]. Recently, three cases of functional E*ML4*-*ALK* fusions with attenuated single orange signals in addition to fusion signals have been analyzed [12], a signal pattern which is quite similar to the pattern of our case.

Both RNA-Seq panels detected three different variants of an *ETV6-NTRK3* fusion. Apart from the dominant classical ETV6:exon5-NTRK3:exon15 variant, which is typical for secretory carcinomas of the salivary gland [9], we found variants ETV6:exon5-NTRK3:exon16 and ETV6:exon4-NTRK3:exon15, albeit both at very low frequency. Several non-classical *ETV6-NTRK3* fusion variants have been described [13, 14], and secretory carcinomas with these variants seem to be more aggressive than tumours with the classical variant, which applies to our case, too. However, to the best of our knowledge co-existence of several variants of the *same* fusion gene in a single tumour has not been published until today. Given the very low abundance of the non-classical variants in our case, a more plausible explanation would be some malfunction of the splicing apparatus, with occasional skipping of exon 5 of ETV6 or skipping of exon 15 of NTRK3 during splicing of the ETV6:exon5-NTRK3:exon15 pre-mRNA.

In summary, the case presented here is particular for two reasons. First, independent NGS analyses with two different panels detected three variants of an *ETV6-NTRK3* fusion gene. However, we hypothesize that only the dominant classical variant ETV6:exon5-NTRK3:exon15 truly exists at the genomic level. The main finding in this case is a very uncommon FISH signal pattern with faint single 3′ signals, which could easily be overlooked in routine diagnostics. It is nevertheless a significant finding of high predictive value, indicating a functional *NTRK3* rearrangement that is successfully targeted by a Trk inhibitor.

## Data Availability

The datasets generated and/or analysed during the current study are not publicly available due to privacy protection.

## Abbreviations

FISH: Fluorescence in situ hybridization
IHC: Immunohistochemistry
NGS: Next-generation sequencing
RNA-Seq: RNA-based next generation sequencing
RT-qPCR: Quantitative reverse transcription PCR
